# Assessment of health-related quality of life in asthmatic patients

**DOI:** 10.1192/j.eurpsy.2023.1896

**Published:** 2023-07-19

**Authors:** A. Omrane, I. Touil, R. Romdhani, Y. Brahem, S. Bouchareb, L. Boussoffara, J. Kneni, T. Khalfallah, N. Keskes

**Affiliations:** 1 Occupational Medicine and Ergonomics; 2 Pneumology department, Faculty of Medicine of Monastir Tunisia, Monastir, Tunisia

## Abstract

**Introduction:**

Asthma is a common worldwide, chronic respiratory disease. It has been shown to impair a person’s health-related quality of life (HR-QoL), but the core influencing factors are not fully understood.

**Objectives:**

We aimed in this study to evaluate QoL of asthmatic patients and its main determinants.

**Methods:**

A prospective single center study was held with asthmatics consulting in the Pulmonology Department a public hospital in Tunisia. A complete structured questionnaire concerning socio-demographic and clinical characteristics were determined. The assessment of asthma control during the last 4 weeks was based on the GINA 2022 report criteria. The quality of life was assessed by the Asthma Quality of Life Questionnaire (AQLQ) scale in its validated Arabic version.

**Results:**

A total of 109 asthmatic patients was included. Most of them were female (N= 73, 67%). Twelve (11%) were current or ex-smokers. The majority of patients (N=101, 92.6%) were active. Thirty-nine patients (35.7%) had comordidities. Asthma were controlled in 40.4% of cases.

The average of AQLQ was4.9±1.2. The most affected domains were environmental stimuli and symptoms with a mean value of 4.6 ± 1.3 and 5.0± 1.3 respectively.

Allergic and uncontrolled asthma and severe disease were significantly associated with the average of AQLQ in the study population with p respectively 0.001, <0.000 and <0.000.

Multivariate analysis demonstrated that factors independently associated with the HR-QoL were : the severity of asthma (OR-0.39, IC95% [-0.62,-0.15], p=0.001) and uncontrolled disease (OR=0.59, IC95%,[-0.87,-0.31], p=0.000).

**Conclusions:**

These results suggest that uncontrolled and severe asthma significantly affect health asthma-related quality of life.

**Disclosure of Interest:**

None Declared

